# Use of Potassium Hydroxide in Dermatology Daily Practice: A Local Study From Saudi Arabia

**DOI:** 10.7759/cureus.30612

**Published:** 2022-10-23

**Authors:** Mahdi Al Dhafiri, Almunthir S Alhamed, Mohammed A Aljughayman, Khurayzan F Bin Sifran, Bashayer F Al Furaikh, Noor Alosaif

**Affiliations:** 1 Dermatology, King Faisal University, Hofuf, SAU; 2 College of Medicine, King Faisal University, Hofuf, SAU; 3 Pediatric Surgery, Maternity and Children Hospital, Dammam, SAU

**Keywords:** daily practice, fungal infection, al-ahsa, dermatologists, koh

## Abstract

Introduction

Potassium hydroxide (KOH) is an inorganic keratolytic test. It is considered one of the most worldwide methods used in the dermatological field with reasonable reliability and sensitivity. One of its major uses is in identifying fungal elements in the skin, hair, nails, and different body secretions. In this study, we aimed to identify the prevalence of KOH usage in different hospitals in the Al-Ahsa region, Saudi Arabia. Additionally, we aimed to identify the dermatological conditions in which KOH is being used and practiced by physicians.

Methods

This was an anonymous questionnaire-based cross-sectional study that was distributed and carried out among dermatologists in Al-Ahsa city in the Eastern province of Saudi Arabia.

Results

A total of 30 dermatologists completed the study questionnaire. Participants’ ages ranged from 29 to 59 years old with a mean age of 39.5 ± 11.4 years old. 14 (46.7%) dermatologists reported that the KOH test is available in their clinic. Using KOH once a day was reported among 14.3% while 21.4% used it once a week. The most reported condition for KOH use was hair dermatophytes (100%).

Conclusion

There is a clear lack of usage and availability of KOH in clinics. The shortage of availability of diagnostic tools, including KOH might affect the diagnosis of several diseases and may cause a waste of resources, wrong prescriptions, and patient’s burden.

## Introduction

Potassium hydroxide (KOH) is considered an inorganic compound in which the chemical formula of it is KOH. It is a renowned keratolytic material, and KOH is frequently used in wet mount preparation [1]. It is considered one of the most global methods used in the dermatological field. One of its major uses is in the diagnosis of fungal elements in the skin, hair, nails, and different body secretions [2]. When discussing the way KOH works, it mainly functions by identifying the fungal structures in keratin cells. To enhance the quality of the result of KOH, a clearing agent should be added, as seen in multiple comparative studies [3]. KOH is a fast, cheap, and most accessible material; however, it does not provide color contrast and demands skillful experts to clarify and read the results [4]. Several studies have compared the efficacy and usage of KOH with other diagnostic tools, and they have revealed that the diagnosis of oral candidiasis is more accurate with the use of fluorescence staining when compared with the usual 10% KOH [5]. Another important example is when evaluating the three main techniques in the diagnosis of pityriasis versicolor, including the microscopic examination using KOH, culture, and molecular methods. While analyzing the results of these methods, Chicago Sky Blue 6B stain is fast, highly sensitive, and better in quality when compared to the culture and KOH mount and is the preferred method of usage in the diagnosis of pityriasis versicolor [6]. Contrary to popular opinion, the sensitivity of Periodic Acid-Schiff (PAS) staining of nail clippings is to some extent higher than KOH and culture when trying to identify onychomycosis [7].

While the clinical significance of KOH has been well established in previous studies, no local or regional articles have discussed its presence and availability in dermatological clinics. In this study, our goal was to identify the prevalence of KOH usage in academic, private, and public hospitals in Al-Ahsa city, Saudi Arabia. In addition, we aimed to examine the dermatological cases in which KOH is being used and practiced by physicians. Further, we tried to identify the probable relationship between the number of years of experience and the sensitivity of reaching an accurate diagnosis without the usage of KOH.

## Materials and methods

This was an anonymous questionnaire-based cross-sectional study that was distributed and carried out among dermatologists in Al-Ahsa city in the Eastern province of Saudi Arabia. The inclusion criteria included: actively practicing dermatologists and currently working in Al-Ahsa city. All physicians were explained the nature and goal of the research and the voluntary nature of participation in the research. With a total of 30 dermatologists interviewed, the sample of the study included physicians from public, private, and academic practices in the city of Al-Ahsa. To protect the confidentiality of respondents, surveys were given an identification code available only to the investigator. The authors constructed an online semi-structured questionnaire that was built on a literature review basis and subject to expert validation. The questionnaire was piloted for validity testing. This study was approved by the institutional review board (IRB) deanship of scientific research at King Faisal University (EA00038) after the related ethical aspects were investigated.

The questionnaire contained 17 questions, which were divided into two main sections. The first section included basic biographical data, which compromised the personal and academic background of the participant. The second section focused on the availability and usage of KOH in dermatological clinics; moreover, it included questions that assess the knowledge about the importance of KOH in diagnosing and treating variant dermatological conditions, the frequency of using KOH in clinical procedures, and the type of dermatological conditions for which the KOH was used.

The questionnaire was distributed manually to the dermatologists, and all the related questions and inquiries have been clarified. After data extraction, they were revised, coded, and fed to statistical software IBM SPSS version 22 (SPSS, Inc. Chicago, IL). All statistical analysis was accomplished using two-tailed tests. P value less than 0.05 was statistically significant. Descriptive analysis based on frequency and percent distribution was done for all variables including personal data, work setting, and years of experience. Additionally, frequency distribution for KOH availability, use, limitations, and diagnostic ability was discussed. Cross tabulation was used to assess the distribution of dermatologist-related personal and experience data by the KOH test’s relevant use and availability. Correlations were tested using an exact probability test for small frequency distributions to assess significance.

## Results

A total of 30 dermatologists completed the study questionnaire. Participants’ ages ranged from 29 to 59 years old with a mean age of 39.5 ± 11.4 years. The population of our sample was distributed as specialists numbered 21 (70%), seven (23.3%) were consultants, and two (6.7%) were dermatology staff physicians. As for additional qualifications, 19 (63.3%) were general dermatologists, eight (26.7%) were cosmetic dermatologists, four (13.3%) were pediatric dermatologists, and three (10%) were immuno-dermatologists. Exactly 10 (33.3%) work at private clinics, nine (30%) at governmental hospitals, nine (30%) at private hospitals, and two (6.7%) at university polyclinics. Considering years of experience, 10 (33.3%) had the experience of fewer than 10 years, while 11 (36.7%) had an experience of more than 20 years. Considering the number of patients treated/seen in the clinic per day, 14 (46.7%) having less than 20 patients daily while 10 (33.3%) care for more than 30 patients (Table 1).

**Table 1:** Personal and professional data of dermatologists in Al-Ahsa, Saudi Arabia

Personal data	No	%
Age in years		
< 40	10	33.3%
40–49	14	46.7%
50+	6	20.0%
Position		
Consultant	7	23.3%
Staff physicians	2	6.7%
Specialist	21	70.0%
Qualifications		
General dermatologist	19	63.3%
Cosmetic dermatologist	8	26.7%
Pediatric dermatologist	4	13.3%
Immuno-dermatologist	3	10.0%
Place of practice		
Governmental hospital	9	30.0%
Private clinic	10	33.3%
Private hospital	9	30.0%
University hospital	2	6.7%
Years of experience		
< 5	3	10.0%
5–10	7	23.3%
11–20	9	30.0%
> 20	11	36.7%
Number of patients treated/seen in the clinic per day		
< 15	5	16.7%
15–20	9	30.0%
21–30	6	20.0%
> 30	10	33.3%

Exactly 14 (46.7%) dermatologists reported that a KOH test is available in their clinic. Using KOH daily was reported by 14.3% while 21.4% used it once a week and 21.4% used it once a month. The most reported conditions for KOH use were hair dermatophytes (100%), skin dermatophytes (83.3%), and nail dermatophytes (75%). Factors that led to unavailability include lack of experience (6.7%), sending samples to a lab (6.7%) with no need to do the test by the physicians themselves, and the non-availability of a microscope (3.3%). Exactly 78.6% reported that they send samples to the lab while 21.4% do the test in the clinic. Also, 42.9% of KOH users reported that KOH examinations usually (80%) fit with their clinical diagnosis, and 35.7% reported often fitting (50%-80%). As for the case of KOH equipment unavailability, 53.3% reported performing clinical examination only, 46.7% sending for culture, and 20% either set to external laboratory or performed a Wood's lamp instead (Table 2).

**Table 2:** Distribution of KOH availability uses and accuracy among dermatologists

		No	%
Is a potassium hydroxide (KOH) test available in your clinic?	Yes	14	46.7%
	No	16	53.3%
If yes, how often do you use it?	Once a day	2	14.3%
	Once every couple of days	2	14.3%
	Once a week	3	21.4%
	Every 2 weeks	2	14.3%
	Once a month	3	21.4%
	I have it in my clinic but I don't use it	2	14.3%
What conditions do you use it for?	Skin Dermatophytes	10	83.3%
	Hair Dermatophytes	12	100.0%
	Nail Dermatophytes	9	75.0%
	Pityriasis Rosea	1	8.3%
If no, what limitations have you experienced?	Available	14	46.7%
	Availability of the microscope	1	3.3%
	Examining by self is a more confident way	1	3.3%
	Good in diagnosis	1	3.3%
	I have not experienced	2	6.7%
	I send the sample to the lab	2	6.7%
	inexperienced lab personnel	1	3.3%
	Need time to do	1	3.3%
	No limitations	1	3.3%
	Not available by the hospital	1	3.3%
	Not available in our clinic	2	6.7%
	Not much	1	3.3%
	Time consuming in rush clinic	1	3.3%
	Very rarely - a non-reliability of test	1	3.3%
Do you do the test in the clinic (KOH)? Or do you send the sample to the laboratory?	Done in the clinic	3	21.4%
	Send sample to the lab	11	78.6%
Is there an available light microscope inside your clinic?	Yes	8	26.7%
	No	22	73.3%
Does the KOH examination fit with your clinical diagnosis?	Rarely (less than 25%)	1	7.1%
	Sometimes (25%-50%)	2	14.3%
	Often (50%-80%)	5	35.7%
	Usually (80%)	6	42.9%
In case of KOH equipment unavailability, what do you do?	Send for culture	14	46.7%
	Perform clinical examination only	16	53.3%
	Send for external laboratory	6	20.0%
	Perform wood's lamp instead	6	20.0%
	Dermatoscopy	3	10.0%

KOH was available at 54.5% of governmental hospital dermatology clinics in comparison to 42.1% of private hospital clinics with no significant difference (P=.510). It was used for skin dermatophytes and hair dermatophytes in all cases in governmental hospitals compared to all hair dermatophytes and 85.7% of nail dermatophytes in private hospitals (P=.475) (Table 3).

**Table 3:** Distribution of KOH availability and uses by work setting

KOH uses	Place of practice				P-value
	Governmental hospital		Private hospital		
	No	%	No	%	
Is a potassium hydroxide (KOH) test available in your clinic?					.510
Yes	6	54.5%	8	42.1%	
No	5	45.5%	11	57.9%	
What conditions do you use it for?					.475
Skin dermatophytes	5	100.0%	5	71.4%	
Hair dermatophytes	5	100.0%	7	100.0%	
Nail dermatophytes	3	60.0%	6	85.7%	
Pityriasis Rosea	0	0.0%	1	14.3%	

All dermatologists who see less than 15 patients daily use KOH once every couple of days. Also, 25% of those who see 15-20 patients use KOH once weekly to once monthly. Exactly 40% of dermatologists who care for more than 30 patients daily use KOH once a week and 20% for different frequencies with no statistical significance (P=.241) (Table 4).

**Table 4:** The relationship between the number of daily patients seen by dermatologists and the frequency of using KOH

Frequency of using KOH	Number of patients treated/seen in the clinic per day									P-value	
	< 15		15-20		21-30		> 30				
	No	%	No	%	No	%	No	%			
Once a day	0	0.0%	0	0.0%	1	25.0%	1	20.0%	.241		
Once every couple of days	1	100.0%	0	0.0%	0	0.0%	1	20.0%			
Once a week	0	0.0%	1	25.0%	0	0.0%	2	40.0%			
Every two weeks	0	0.0%	0	0.0%	1	25.0%	1	20.0%			
Once a month	0	0.0%	1	25.0%	2	50.0%	0	0.0%			
I have it in my clinic but I do not use it	0	0.0%	2	50.0%	0	0.0%	0	0.0%			

About 66.6% of dermatologists with experience of fewer than five years usually reach an accurate diagnosis without the use of KOH compared to 45.5% of those with experience years exceeding 20 years. On the other hand, 22.2% of dermatologists with experience of 11-20 years reach an accurate diagnosis without the usage of KOH compared to 18.2% of those with experience exceeding 20 years (Table 5).

**Table 5:** The relationship between the number of years of experience and reaching an accurate diagnosis without the use of KOH

Accurate diagnosis without the use of KOH	Years of experience								P-value		
	< 5		5-10		11-20		> 20				
	No	%	No	%	No	%	No	%			
Rarely (less than 25%)	1	11.1%	0	0.0%	0	0.0%	0	0.0%	.351		
Sometimes (25%-50%)	0	0.0%	2	28.6%	1	11.1%	0	0.0%			
Often (50%-80%)	1	33.3%	1	14.3%	0	0.0%	4	36.4%			
Usually (80%)	2	66.7%	4	57.1%	6	66.7%	5	45.5%			
Always (100%)	0	0.0%	0	0.0%	2	22.2%	2	18.2%			

## Discussion

Potassium hydroxide (KOH) is considered to be a well-known examination that is used in a variety of cutaneous pathological conditions. The role of KOH is well established in dermatological guidelines and scientific articles [8,9]. One of the published studies about different diagnostic methods that are used in a variety of cutaneous conditions has shown that KOH preparation along with fungal cultures is the two most important methods in diagnosing fungal nail infection [10]. As an example, a previous study conducted in the US that aimed to identify and evaluate physician’s preferences and uses of diagnostic tests for toenail onychomycosis included 1,000 randomly sampled physicians from three different specialties, dermatologists, podiatrists, and family practitioners and revealed that KOH was the preferred diagnostic test for all of them [11]. However, a controversy about the actual practical use of KOH among dermatologists is not yet documented according to our knowledge. In our local study, we have looked into this aspect with practicing dermatologists and tried to unravel the current reality of KOH use in Al-Ahsa Saudi Arabia.

The mean age for the participant dermatologists was 39.5 years, which coincides with other cross-sectional studies conducted among dermatologists, and reflects the median age of physicians in Saudi Arabia [12,13]. The study targeted high-exposure physicians as 83.3% of dermatologists see more than 15 patients per shift, which is higher than in other settings and presents as a burden on physicians which may lower their productivity and efficacy at work, including the low number of requests for KOH tests [14].

Even though KOH has a significant value, less than 50% of dermatologists in Al-Ahsa reported its presence in the clinics. While we were comparing the accessibility of KOH between governmental and private clinics, we noticed that about 54% of government clinics confirmed the presence of KOH with only 42% in private practice. This is a major barrier and an important obstacle as the use of any other alternative or referral is not as convenient as the presence of this modality in the clinic. As a consequence of low availability, only 14% of dermatologists use KOH daily. Furthermore, 21% of dermatologists use KOH once monthly, which approximately indicates that KOH is used for one patient among every 300 patients that the dermatologists see. This can have different explanations, including the unavailability of KOH or that physicians do not have the time to use this tool for every patient, the physician relying on his/her own expertise, having a non-experienced microbiologist, poor sample collection from the patient, or even because dermatologists do not appreciate the clinical importance of using KOH. As a result, more than half of the samples (53.3%) were confined to clinical examination alone, with no further confirmatory measures. Additionally, only 20% send the patient to external laboratories.

The majority of the interviewed dermatologists agreed on the high value of the usage of KOH in dealing with variant types of dermatophytes from skin (100%), hair (83.3%), and nails (75%). This emphasizes the significant value of using this modality daily in dermatological clinics, especially while several studies have stated that the use of KOH is essential for the diagnosis of tinea capitis [15,16]. About half of the respondents (42.9%) stated that their clinical diagnosis usually fits the actual result of KOH, while (35.7%) reported that their clinical diagnosis often fits the actual result of KOH. This demonstrates that a significant number of patients need a KOH examination to reach an accurate diagnosis.

Regarding the relationship between the number of years of experience and the ability to reach an accurate diagnosis without the use of KOH, only 45.5% of dermatologists with experience exceeding 20 years feel confident to reach the correct diagnosis without KOH use compared to 22% who have an experience between 11 and 20 years. This subjective confidence is confirmed by follow-up visits as well as post-therapeutic results. Surprisingly, 66% of dermatologists with an experience of fewer than five years claim that they can reach an accurate diagnosis without the help of KOH.

We are aware of several limitations to our article; one example is the small sample size of participants. Additionally, it is expected that the frequency of use of the KOH test is variable due to variability in the daily cases; therefore, the number provided by the participants regarding the frequency of the KOH test is approximate. Moreover, the confirmation of the diagnosis with those who do not practice laboratory-diagnostic tools is clinical and dependent on the outcome after the antifungal treatment.

## Conclusions

Our study shows a general consensus on the importance and value KOH provides to dermatologists for diagnosing various disorders. However, there is an obvious shortage of both the presence of KOH in clinics and its availability elsewhere. This should prompt health authorities to invest more in providing easy accessibility of this tool to dermatologists. Furthermore, our study shows that arranging for a reasonable workload and decreasing the number of patients seen by dermatologists enhanced the overall care and allowed physicians to confirm their diagnoses using KOH. Future studies should aim to understand and compare the reality of the use of KOH in different areas and at both national and regional levels.
